# Metabolism of Exogenous [2,4-^13^C]β-Hydroxybutyrate following Traumatic Brain Injury in 21-22-Day-Old Rats: An Ex Vivo NMR Study

**DOI:** 10.3390/metabo12080710

**Published:** 2022-07-29

**Authors:** Susanna Scafidi, Jennifer Jernberg, Gary Fiskum, Mary C. McKenna

**Affiliations:** 1 Department of Anesthesiology and Critical Care Medicine, Johns Hopkins University School of Medicine, Baltimore, MD 21287, USA; sscafid2@jhmi.edu (S.S.); jjernb@lsuhsc.edu (J.J.); 2 Department of Anesthesiology, Center for Shock Trauma and Anesthesiology Research, University of Maryland School of Medicine, Baltimore, MD 21201, USA; gfiskum@som.umaryland.edu; 3 Department of Pediatrics, University of Maryland School of Medicine, Baltimore, MD 21201, USA; 4 Program in Neuroscience, University of Maryland School of Medicine, Baltimore, MD 21201, USA

**Keywords:** traumatic brain injury, developing brain, ketone bodies, metabolism, β-hydroxybutyrate

## Abstract

Traumatic brain injury (TBI) is leading cause of morbidity in young children. Acute dysregulation of oxidative glucose metabolism within the first hours after injury is a hallmark of TBI. The developing brain relies on ketones as well as glucose for energy. Thus, the aim of this study was to determine the metabolism of ketones early after TBI injury in the developing brain. Following the controlled cortical impact injury model of TBI, 21-22-day-old rats were infused with [2,4-^13^C]β-hydroxybutyrate during the acute (4 h) period after injury. Using ex vivo ^13^C-NMR spectroscopy, we determined that ^13^C-β-hydroxybutyrate (^13^C-BHB) metabolism was increased in both the ipsilateral and contralateral sides of the brain after TBI. Incorporation of the label was significantly higher in glutamate than glutamine, indicating that ^13^C-BHB metabolism was higher in neurons than astrocytes in both sham and injured brains. Our results show that (i) ketone metabolism was significantly higher in both the ipsilateral and contralateral sides of the injured brain after TBI; (ii) ketones were extensively metabolized by both astrocytes and neurons, albeit higher in neurons; (iii) the pyruvate recycling pathway determined by incorporation of the label from the metabolism of ^13^C-BHB into lactate was upregulated in the immature brain after TBI.

## 1. Introduction

### 1.1. Traumatic Brain Injury

Traumatic brain injury (TBI) remains the leading cause of mortality and morbidity in children in the US, where annually, approximately 500,000 children sustain TBI, resulting in about 35,000 hospitalizations and 3000 deaths per year [1]. Pediatric TBI can lead to poor neurodevelopmental outcomes since the injury is superimposed on the rapidly developing brain, which has a high requirement for energy metabolism to support synaptogenesis, myelination, and biosynthesis of macromolecules [2]. Survivors of pediatric brain trauma often suffer from life-long disabilities, including severe cognitive and motor delays and psychosocial impairment [3,4,5,6].

Glucose is the primary substrate for the brain’s high energy demands; however, following TBI, oxidative glucose metabolism is dysregulated, resulting in a ‘metabolic crisis’ characterized by an elevated lactate/pyruvate ratio in microdialysis of TBI patients despite normal brain oxygen levels [7]. Both clinical reports [8] and studies with animal models [9] show impairment in brain glucose metabolism after TBI not only at the site of the focal cortical injury but also in the hippocampi bilaterally, which is associated with cell death and long-term volume loss [10,11,12,13]. Previous preclinical reports demonstrated that oxidative glucose metabolism is reduced within several hours after TBI [10,11,12]. Oxidative damage to the pyruvate dehydrogenase enzyme complex, which is known to occur after acute brain injury, can decrease the conversion of pyruvate from glucose metabolism to acetyl CoA and thus lead to less oxidative metabolism via the tricarboxylic acid (TCA) cycle [11,14]. It has been reported that, following TBI in the adult rat brain, there is an acute period of cerebral glucose hypermetabolism, which is short-lived, and subsequently followed by cerebral metabolic depression, which starts at 6 h and lasts for days [15]. Similar to adults, after TBI, 17-day-old rats exhibited acute hypermetabolism in the brain with subsequent cerebral metabolic depression, but the timing of this switch is not clearly defined [16]. Our group previously observed both decreased and delayed metabolism of [1,6-^13^C]glucose between 5 and 6 h after TBI in 20–21-day-old rats [11]. Delayed incorporation of glucose metabolism into glutamate, glutamine, and gamma-aminobutyric acid (GABA) after injury and the labeling patterns showed evidence of impaired metabolism both via pyruvate dehydrogenase and the astrocyte-specific pyruvate carboxylase pathway [11]. Robertson et al. [17] showed that pyruvate dehydrogenase complex activity was lower in mitochondria at 4 h after TBI in immature (postnatal day 17) rat brain. These results suggest that cerebral metabolic depression in the developing brain may begin earlier than 6 h; thus, alternative substrates capable of supporting oxidative metabolism should be administered within the first few hours after TBI.

### 1.2. Role of Ketones in Brain Energy and Metabolism after Traumatic Brain Injury

It is well-established that the brain is metabolically flexible and can metabolize other substrates, such as the ketone bodies—acetoacetate and β-hydroxybutyrate—in addition to glucose [18]. Numerous studies have demonstrated that ketones are important substrates in the developing brain [2,18]. Ketones are metabolized by both the developing and adult brain, especially under pathological conditions, such as starvation. Ketogenic diets are currently used as non-pharmacological therapy for intractable epilepsy in children, and especially introduced early in cases of glucose transporter 1 deficiency syndrome, pyruvate dehydrogenase deficiency, and complex I mitochondrial disorders [19]. There are several mechanisms by which ketones may affect brain metabolism [20]. Ketones have been reported to modify histone deacetylase (HDAC) inhibitors [21,22], alter the activity of K^+^-ATP channels [23], the regulation of Ca^2+^ channels [24], and increase mitochondrial succinate levels [25], thus increasing metabolism via complex II of the electron transport chain. Ketones and/or a ketogenic diet have been shown to be neuroprotective in a number of studies after brain injury (see extensive reviews [26,27]). Ketones have been shown to decrease lesion volume, improve ATP production, decrease cell death and increase cell survival, decrease edema and lipid peroxidation, decrease oxidative stress, improve mitochondrial respiratory complex activity, and improve neurological outcome [26,27,28]. Despite numerous *in vitro* and *in vivo* studies reporting positive effects of ketones, studies on TBI are limited and primarily focused on the effects of a ketogenic diet after TBI [26,27] or fasting [29]. To date, there are few, if any, reports of the effects of ketones in a model of pediatric TBI [30,31].

Most of these studies have been based on the premise that β-hydroxybutyrate and acetoacetate can directly provide an acetyl-CoA moiety for metabolism via the TCA cycle, thus bypassing the pyruvate dehydrogenase complex [2,20,26,27]. Since TBI is characterized by decreased activity of pyruvate dehydrogenase—a key enzyme that links glycolysis and the TCA cycle—resulting in lactic acidosis and disrupted oxidative metabolism, ketones, which enter metabolism at the level of acetyl CoA, can sustain oxidative metabolism [14]. Recently, Greco and colleagues reported that administration of β-hydroxybutyrate to young adult male rats during the first 3 h post-injury resulted in increased levels of acetyl-CoA in the pericontusional ipsilateral cortex and improved “astrocytic health” at 24 h after TBI [32]. However, to date, there is no direct evidence that an injured brain is able to metabolize ketones for energy and neurotransmitter synthesis following TBI.

The primary aim of this study was to determine whether β-hydroxybutyrate (BHB) can directly fuel mitochondrial brain energy metabolism after moderate–severe brain trauma in the immature rat model of pediatric TBI. Specifically, we aimed to determine the metabolism of [2,4-^13^C]β-hydroxybutyrate in the injured brain in a clinically relevant setting. Several aspects of the experimental design increased the clinical relevance of this study, including (1) rats were not fasted prior to surgery (TBI injury); (2) BHB was administered intravenously after TBI since food is withheld from patients in the first hours after injury to closely monitor neurologic status; and (3) an isonatremic concentration of BHB was administered.

## 2. Materials and Methods

### 2.1. IACUC Statement

All procedures were performed in accordance with the National Institute of Health’s Guide for the Care and Use of Laboratory Animals and under the approval of the Animal Care and Use Committee at the University of Maryland School of Medicine.

### 2.2. Biochemicals

The sodium [2,4-^13^C] β-hydroxybutyrate (CLM-3706-PK, 99% ^13^C enriched) and sodium 3-(trimethylsilyl)propionate-2,2,3,3-d4 (TMSP, DLM-48-1, 98%) were purchased from Cambridge Isotope Laboratories, Woburn, MA, USA. Dioxane (D111-500, 99.9%) was obtained from Thermo Fisher Scientific, Waltham, MA, USA. Perchloric acid (244252-500 ML, 70%) was obtained from Sigma-Aldrich, St. Louis, MO, USA. Pierce BCA Protein Assay kit was purchased from ThermoFisher, Waltham, MA, USA (cat.#23227).

### 2.3. TBI Model: Controlled Cortical Injury

**Figure 1:**
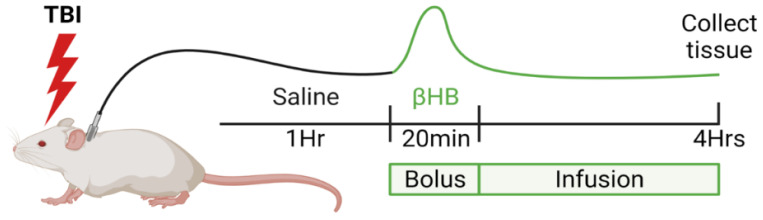
Experimental paradigm of infusion of β-hydroxybutyrate (BHB) following sham or TBI surgery. Figure was created using BioRender.com (accessed on 26 May 2022).

Experiments were conducted using immature postnatal day 21–22 (PND 21–22) Sprague Dawley male rat pups (total number of animals used n = 44) weighing 45–55 g (Charles River Laboratories, Wilmington, MA, USA). All animals were weaned from lactating dam 48 h prior to the experiment (PND19–20) and housed in large shoebox-type cages n = 3/cage under standard conditions: 12 h/12 h lighting cycle (07:00–19:00, temperature 23 ± 2 °C, relative humidity 65%, with minimum noise and handling. All animals had free access to standard chow diet (2018SX, Teklad Global, Indianapolis, IN, USA) and water up until the time of surgery. Rat pups were anesthetized with isoflurane (4% induction and 1.75% maintenance via nose cone during the entire surgical procedure). The right femoral vein was catheterized using 1Fr catheter (Instech Solomon, Plymouth Meeting, PA, USA), threaded subcutaneously to the shoulder blades and attached to a swivel for infusion. Traumatic brain injury was performed using the control cortical impact (CCI) model as previously described [11]. In brief, following left parietal craniotomy, moderate–severe TBI was performed using a controlled cortical impact device (1.5 mm depth of penetration, 5.0 ± 0.3 m/s velocity) (Pittsburgh Precision Instruments, Pittsburgh, PA, USA). Sham animals had craniotomy only. Following injury, the bone flap was replaced, craniotomy sealed, and the scalp incision closed with sutures. Anesthesia was discontinued; animals regained consciousness and remained individually housed for the entire duration of experiment with free access to water and no access to food. During the first hour post-surgery, animals received infusion of normal saline (NS), followed by a bolus of 200 mM [2,4-^13^C]β-hydroxybutyrate (BHB) (Cambridge Isotope Laboratories, Andover, MA, USA) over 20 min, followed by continuous infusion of 30 mg/kg/h BHB until 4 h post-injury (modified from Pan et al. [33]). The sham and TBI animals were awake and freely moving in clear enclosure (stank, Instech Solomon, Plymouth Meeting, PA, USA) after surgery throughout the infusion period. Point-of-care blood levels of glucose and β-hydroxybutyrate were measured using Precision Xtra meter (Abbott, Alameda, CA, USA). Blood samples were obtained via minimally invasive tail-prick method, with several measurements taken from the same animals during the time-course data collection. At 4 h post-TBI, rats were euthanized, brains were rapidly dissected into injured (left, ipsilateral (ipsi)) and contralateral (right, contra) sides that included the injured area of cortex and underlying hippocampus, and snap-frozen in liquid nitrogen (Figure 1). Blood samples were collected, and the plasma was separated by centrifugation (3000 rpm, 10 min, 4 °C). All samples (brain and plasma) were stored at (−80 °C) until further NMR extraction.

### 2.4. Immunohistochemistry

A separate group of animals underwent craniotomy and the TBI procedure and were infused with unlabeled 0.2 M β-hydroxybutyrate (BHB) (Sigma, Cat# 54965) (see surgery description as above). At 4 h post-TBI, animals were anesthetized with isoflurane and transcardially perfused with 0.9% saline (*w*/*v*), followed by the perfusion with fixative (4% paraformaldehyde in a phosphate buffer solution (PBS), pH 7.4). The brains were harvested, post-fixed overnight, and subsequently rehydrated with 30% sucrose in PBS. Free-floating 50 µm coronal brain sections were blocked for 1 h and 15 min in 20% natural goat serum, 1% bovine serum albumin, and 0.3% TX-100 in PBS. Sections were incubated with primary antibodies overnight at 4 °C; antibodies included Anti-BDH1 1:500 (Sigma; Cat# HPA030947, Lot R32980), Anti-NeuN 1:1,000 (Millipore, Burlington, MA, USA; Cat# MAB377, Lot 2140038), Anti-GFAP 1:750 (Abcam, Cambridge, MA, USA; Cat# ab4674, Lot GR1473-16). Secondary antibodies raised in goat (FITC anti-rabbit, TRITC anti-mouse, and Alexa Fluor 647 anti-chicken) were applied for 1.5 h at 1:200. Tissue was mounted using Prolong Gold with DAPI (Molecular Probes, Waltham, MA, USA; Cat# P36935). Images were obtained using a Zeiss LSM 510 Meta confocal microscope.

### 2.5. Tissue and Plasma Extraction

All frozen samples were homogenized using 2 mL of 7% ice-cold perchloric acid (PCA), centrifuged for 10 min at 4 °C and 4000× *g* [11]. The supernatants were transferred into the new tubes, the precipitates were re-extracted, and supernatants were combined. The tubes were kept on ice at all the times. The combined supernatants were centrifuged again for 10 min at 4 °C and 4000× *g*. The PCA extracts were neutralized with KOH to pH 7, centrifuged, and the supernatant shell frozen and lyophilized. Plasma samples (typically 0.5 mL) were extracted similar to brain samples with 1 mL ice-cold 7% PCA, neutralized, and lyophilized. Lyophilized extracts were stored at −80 °C and reconstituted in D2O with 0.4% dioxane and 0.02% TMSP added as internal standards. The pellets were used for protein concentration using the Pierce protein assay.

### 2.6. NMR Spectroscopy

All spectra were obtained using a Varian Inova 500 MHz spectrometer with a broad band detection probe. The ^13^C-NMR spectra were acquired at 25 °C using a 35° pulse angle, with a 1.3 s acquisition time and 0.5 s relaxation delay. The number of scans was typically 15,000. Some spectra were also broad band decoupled only during acquisition and accompanied by relaxation delay of 20 s to achieve fully relaxed spectra without nuclear Overhauser effects. Correction factors were obtained from the 20 s relaxation delay spectra and applied to the integrals of the individual metabolite peaks to correct for nOe and relaxation delay [34]. The total amount of ^13^C in the resonance of a particular metabolite was calculated using the dioxane (peak at 67.4 ppm) as an internal concentration standard to calculate the nmol ^13^C incorporated per mg protein. Data are expressed as nmol ^13^C incorporated/mg of total protein. The ^1^H-NMR spectra were acquired with a 90° pulse angle, relaxation delay of 11 s, acquisition time of 3.68 s, spectral width 12 parts per million (p.p.m.), 256 scans, and 32 K memory size. Chemical shifts for the ^1^H-NMR spectra determined relative to the TMSP peak at 0.0 ppm. Spectra were analyzed using MestreNova software version 4.1.2 (Mestrelab Research S.L., Madrid, Spain). Enrichment of brain metabolites with ^13^C was calculated as previously described [11].

### 2.7. Statistical Analysis

All data analyses were performed using GraphPad Prism software version 9.2.0 (GraphPad Software, San Diego, CA, USA). All values are reported as the mean ± SEM. Two-way ANOVA was used to compare values from ipsilateral and contralateral sides of injured and sham brain, followed by Bonferroni *post hoc* analysis. A value of *p* < 0.05 was considered significant.

## 3. Results

### 3.1. Infusion of β-Hydroxybutyrate Results in Increased Blood Levels

Following TBI, the blood glucose levels were not different between the groups—F(3,24) = 1.33; *p* = 0.29—but changed over time—F(2.273, 54.55) = 27.52; *p* < 0.001 (Table 1; n = 6–8/group). At 1 h after TBI injury, the blood levels of glucose were increased in all the groups, which can be explained by recovery from anesthesia and a systemic response to the surgery; these levels declined by the end of the experiment (4 h after sham or TBI surgery). Animals treated with exogenous infusion of BHB had significantly higher levels compared to vehicle (saline) treated rats (F(3,20) = 7.35, *p* < 0.01) and changed over time—F(1.69, 33.84) = 3.56, *p* < 0.05.

At 4 h after injury, the blood levels of BHB were 2.15 ± 0.76 mmol/L (mean ± SEM) in sham + BHB animals, which was comparable to the level of 2.65 ± 1.2 mmol/L in the TBI + BHB rats, *p* > 0.9999. Both the sham and TBI animals had comparable blood glucose levels (10.03 ± 1.21 and 8.10 ± 1.92 mmol/L (mean ± SEM)) for sham and TBI, respectively (*p* = 0.22; Table 1; n = 6–8/group).

### 3.2. β-Hydroxybutyrate Dehydrogenase Is Found in Both Neurons and Astrocytes

**Figure 2:**
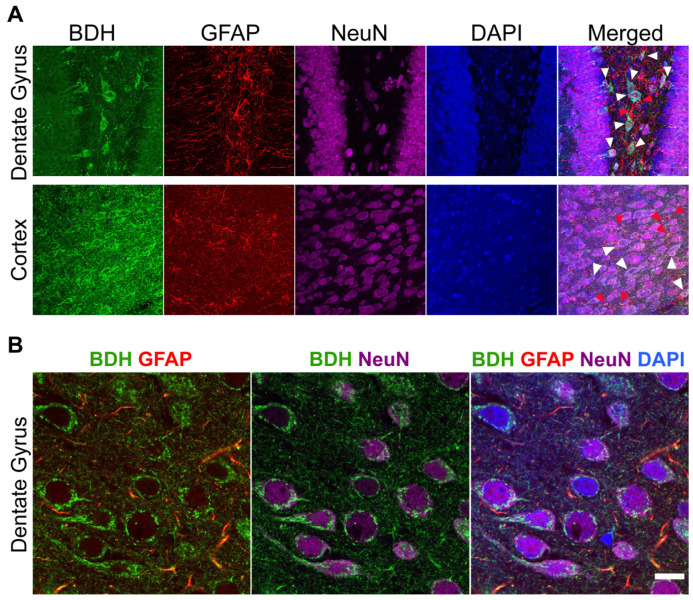
β-hydroxybutyrate dehydrogenase (BDH) labeling is present in both neurons and astrocytes in 21-22-day-old rat brain. (**A**). Representative fluorescence image of BDH staining in brain depicting expression of BDH in both astrocytes (GFAP) and neurons (NeuN). Examples of co-localization of BDH with GFAP are indicated with red arrows and BDH with NeuN with white arrows in the dentate gyrus and cortex. (**B**). High magnification (100×) in the dentate gyrus further illustrates co-localization. Scale bar = 10 µm.

Immunohistochemical analysis of β-hydroxybutyrate dehydrogenase (BDH), a key enzyme in ketone metabolism, revealed that this enzyme was present in both neurons and astrocytes. Labeling of BDH was colocalized with the neuronal marker—neuronal nuclear protein (NeuN) and the astrocyte marker glial fibrillary acidic protein (GFAP) (representative fluorescence images in Figure 2A,B). We also examined whether β-hydroxybutyrate dehydrogenase changes were present early after traumatic brain injury (4 h after CCI TBI). There were no differences in the amount of labeling in the ipsilateral (injured) and contralateral sides of cortex and hippocampus from TBI rat pups compared to shams (data not shown).

### 3.3. Metabolism of [2,4-^13^C] β-Hydroxybutyrate in Brain after TBI

Since β-hydroxybutyrate was infused systemically, we determined ^13^C-metabolites in plasma to ensure that the BHB was not converted to glucose or lactate by other tissues (e.g., heart, muscle). Following the infusion of [2,4-^13^C]β-hydroxybutyrate, there were no differences in plasma ^13^C-BHB between sham and TBI rats (Supplemental Data).

**Figure 3:**
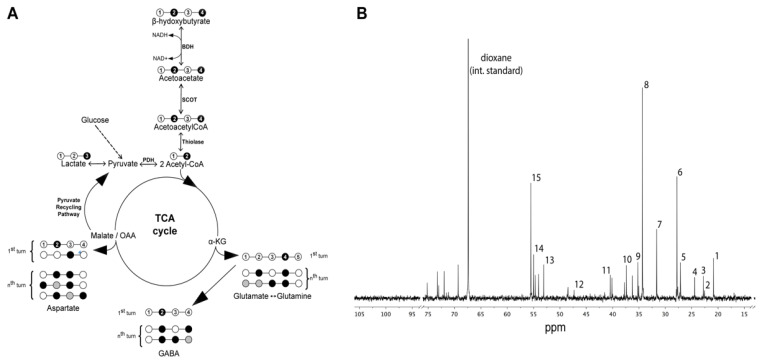
Metabolism of [2,4-^13^C]β-hydroxybutyrate (^13^C-BHB) in the brain. (**A**). Labeling pattern from the metabolism of [2,4-^13^C]β-hydroxybutyrate (^13^C-BHB). (**B**). Typical ^13^C-NMR spectra of rat brain after the infusion of 0.2 M [2,4-^13^C]β-hydroxybutyrate (^13^C-BHB). Peak assignment: (1) lactate C3; (2) BHB C4; (3) N-acetylaspartate C6; (4) GABA C3; (5) Gln C3; (6) Glu C3; (7) Gln C4; (8) Glu C4; (9) GABA C2; (10) aspartate C3; (11) GABA C4; (12) BHB C2; (13) aspartate C2; (14) Gln C2; (15) Glu C2. Abbreviations: glutamate = Glu; glutamine = Gln; BHB = β-hydroxybutyrate.

The labeling pattern from metabolism of [2,4-^13^C]β-hydroxybutyrate in the brain is shown in Figure 3A.

BHB is metabolized in both neurons and astrocytes in the brain. After uptake into brain, [2,4-^13^C]β-hydroxybutyrate is converted to [2,4-^13^C]acetoacetyl CoA, then to [2-^13^C]acetyl CoA. Acetyl CoA labeled in the C2 position enters the TCA cycle, giving rise to [4-^13^C]α-ketoglutarate (α-KG C4), which can be converted to [4-^13^C]glutamate (GLU C4). Subsequently, [4-^13^C]Glutamate can be converted to [2-^13^C]GABA in neurons, or can be converted to [4-^13^C]glutamine (GLN C4) in astrocytes; [4-^13^C]α-KG further metabolized via the TCA cycle gives rise to [2/3-^13^C]succinate due to randomization of the label, and subsequently gives rise to [2/3-^13^C]malate and [2/3-^13^C]oxaloacetate (OAA), which can be converted to [2/3-^13^C]aspartate (ASP C2 & C3). Metabolism in the second and third turns of the TCA cycle leads to labeling in the C3 and C2 positions of glutamate and glutamine, respectively.

The infused [2,4-^13^C]β-hydroxybutyrate was metabolized in the brain of both sham and TBI rats in the presence of normal to slightly elevated plasma glucose levels. A typical ^13^C-spectrum of perchloric acid extract of brain is shown in Figure 3B.

**Figure 4:**
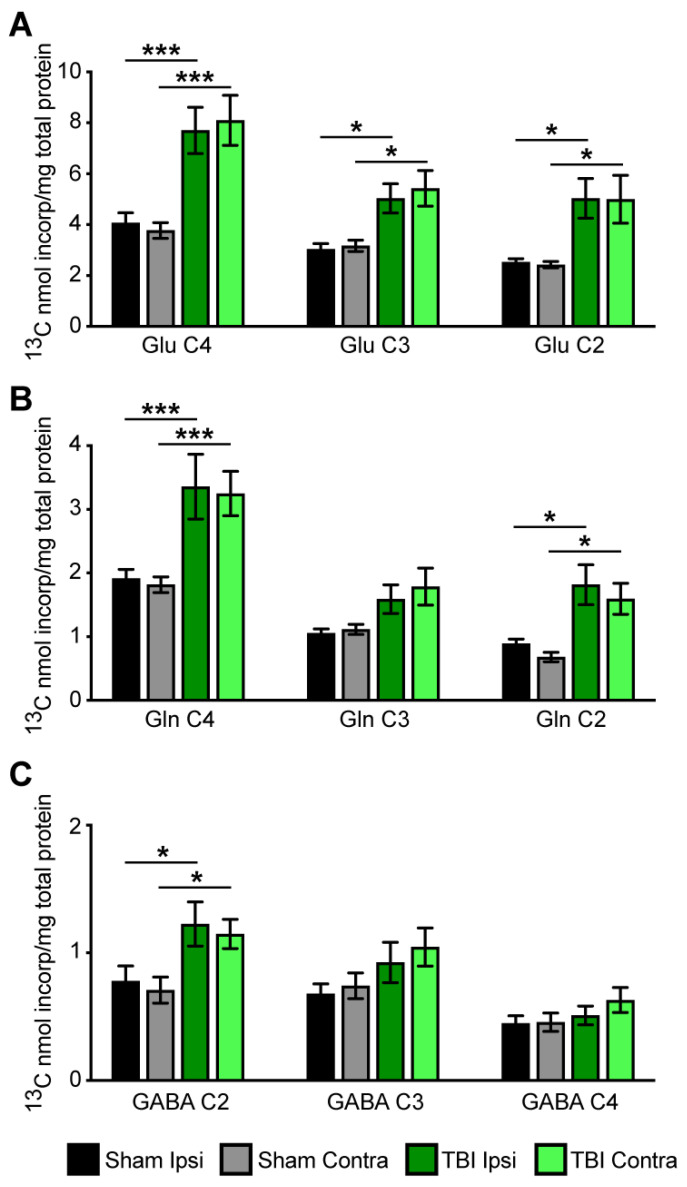
Incorporation of label from the metabolism of [2,4-^13^C]β-hydroxybutyrate into specific carbons (isotopomers) of (**A**) glutamate (Glu), (**B**) glutamine (Gln), and (**C**) GABA in ipsilateral and contralateral cortico-hippocampal formations in sham (grey bars) and TBI-operated (green bars) animals. Values are mean ± SEM nmol ^13^C incorporated/total mg of protein; n = 8 sham and n = 8 TBI. Data were analyzed by 2-way ANOVA with Bonferroni *post-hoc* analysis. *** *p* < 0.001; * *p* < 0.05.

Labeling of metabolites was considerably higher in both the ipsilateral and contralateral sides of TBI brain than in the ipsilateral and contralateral sides of sham animals (Figure 4A–C). Incorporation of label into the C4, C3, and C2 positions of glutamate was considerably higher in both the ipsilateral and contralateral sides of the brain of TBI rats than in shams, as shown in Figure 4A. The nmol ^13^C incorporated/mg protein in the C4 position was significantly higher in both the ipsilateral and contralateral sides of brain from TBI rats compared to shams (F(1, 28) =19.88, *p* < 0.001. Labeling in the C3 and C2 positions of glutamate (Figure 4A) was significantly higher in both the ipsilateral and contralateral sides of the brains from TBI rats compared to sham brains—F(1, 28) = 9.57; *p* < 0.02 and F(1, 28) = 16.94; *p* < 0.003, respectively.

Incorporation of the label into the C4 and C2 positions of glutamine was significantly higher in both the ipsilateral and contralateral sides of the brain of TBI rats than in shams, as shown in Figure 4B. Incorporation of the label into the C2 position of GABA was significantly higher in both the ipsilateral and contralateral sides of the brain of the TBI rats than in shams, as shown in Figure 4C.

We determined the ratio of GLU/GLN in the C4, C3, and C2 positions since the rate of metabolism of BHB in astrocytes and neurons could be different in TBI rats compared to shams. The ratio of labeling in GLU C4/GLN C4 was >2 in the brains from both TBI and sham rats, indicating that metabolism of BHB was higher in neurons than in astrocytes and was not changed by injury, as shown in Table 2.

**Figure 5:**
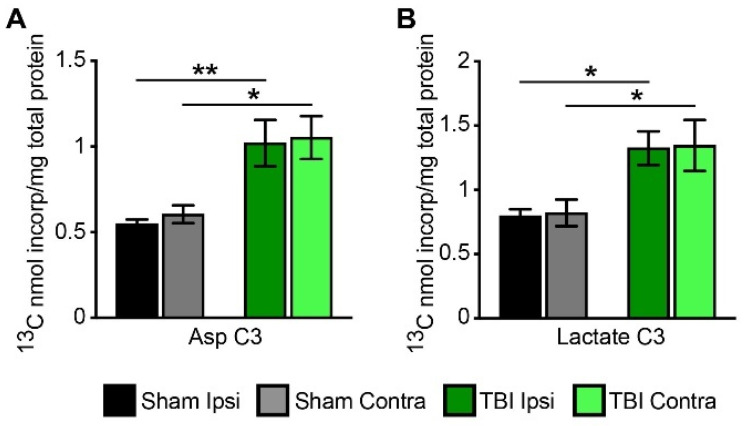
Incorporation of label from metabolism of [2,4-^13^C]β-hydroxybutyrate (^13^C-BHB) into aspartate (Asp C3) (**A**) and lactate (Lac C3) (**B**) in ipsilateral and contralateral cortico-hippocampal formations in sham and TBI-operated animals. Values are mean ± SEM nmol ^13^C incorporated/total mg of protein; n = 8 sham and n = 8 TBI. Data were analyzed by 2-way ANOVA with Bonferroni *post-hoc* analysis. * *p* < 0.05, ** *p* < 0.01.

The incorporation of the label from the metabolism of [2,4-^13^C]β-hydroxybutyrate into the C3 position of aspartate was increased in both the ipsilateral and contralateral sides of the brain from TBI rats compared to shams; F(1, 28) = 22.68, *p* < 0.007 for TBI ipsi vs. sham ipsi, and F(1, 28) *p* < 0.001 for TBI contra vs. sham contra (Figure 5A).

### 3.4. Cycling Ratios

Since TBI is characterized by impaired mitochondrial function, we determined TCA cycle activity by calculating cycling ratios of glutamate, glutamine, and GABA, which are derived from TCA cycle intermediates. Since the C4 position of glutamate is labeled in the first turn of the TCA cycle while C3 and C2 are labeled from subsequent turns, the cycling ratios were calculated in order to evaluate TCA cycle metabolism (e.g., how fast metabolism is taking place in the brains of TBI rats compared to shams) (Table 2). None of the cycling ratios were different in the TBI brains compared to shams, demonstrating that there was no delay in TCA cycle metabolism in the early hours after TBI and metabolism was comparable to sham-operated animals.

Pyruvate recycling has been shown to occur in the brain; in this pathway, malate or oxaloacetate exits the TCA cycle and is converted to pyruvate via either malic enzyme or the combined action of pyruvate kinase and phosphoenolpyruvate carboxykinase (PEPCK) (Figure 2A) [35,36,37]. We assessed the extent of pyruvate recycling by determining the incorporation of the label from the metabolism of [2,4-^13^C]β-hydroxybutyrate into the C3 position of lactate (Lac C3). Both the ipsilateral and contralateral sides of the TBI brain had increased incorporation of label into the C3 position of lactate (Figure 5B); F(1, 28) *p* < 0.04 for TBI ipsi vs. sham ipsi, and F(1, 28) *p* < 0.03 for TBI contra vs. sham contra. These results show that significantly more pyruvate recycling occurred in the brains of TBI rats compared to shams.

## 4. Discussion

In this study, we employed a well-established and widely used model of focal moderate–severe controlled cortical impact (CCI) brain injury, which has been extensively used for decades in TBI research [38]. A single focal impact recapitulates acute and long-term pathological changes in the rodent brain, similar to the changes observed in humans after TBI [39,40]. In the present study and our previous study on glucose metabolism [11], we found that TBI leads to significant changes in metabolism in both the ipsilateral and contralateral sides of the brain compared to shams. Similarly, glucose uptake determined by PET imaging was decreased in both the ipsilateral and contralateral hemispheres of adult rat brain after TBI injury [41]. 

Data from the present study demonstrate that metabolism of [2,4-^13^C]β-hydroxybutyrate was increased in both sides, i.e., ipsilateral and contralateral cortico-hippocampal formations, during the first hours after TBI in the immature 21-22-day-old rat brain. This is in contrast to earlier studies from our group, which found that oxidative glucose metabolism was delayed and dysregulated in both the ipsilateral and contralateral sides of the brain at 5–6 h after TBI in 21-22-day-old rats [11]. Both the labeling of glutamate from neuronal-specific metabolism of glucose and metabolism of glucose via the pyruvate carboxylase pathway in astrocytes were delayed in both sides of the brain after TBI compared with sham brain [11].

It is well-established that, following TBI, oxidative glucose metabolism is dysregulated, resulting in a ‘metabolic crisis’ characterized by an elevated lactate/pyruvate ratio in microdialysis of TBI patients despite normal brain oxygen levels [7]. Both clinical reports [8] and studies with animal models [9] show impairment in brain glucose metabolism after TBI. Preclinical studies showed impairment in glucose metabolism occurred at the site of the focal cortical injury and also involved the contralateral side of the brain and both hippocampi [10,11,12,13]. The increased cell death observed within hours after TBI in preclinical studies and increased loss of brain volume is attributed in part to this early metabolic dysregulation [42,43,44]. Oxidative damage to the pyruvate dehydrogenase enzyme complex (PDH), which occurs after acute injury in immature brain, can decrease the conversion of pyruvate from glucose metabolism to acetyl CoA and thus result in diminished oxidative metabolism via the TCA cycle [11,14]. It is important to note that the developing brain has high metabolic requirements and, therefore, is particularly susceptible to damage from even transient energy deficits [2]. Ketone bodies enter metabolism after the vulnerable PDH step and lead to formation of acetyl CoA, which enters the TCA cycle for oxidative metabolism. Thus, ketones could be valuable therapeutic substrates for maintaining oxidative energy production in the immature brain early after TBI when glucose metabolism is dysregulated.

The increased [2,4-^13^C]β-hydroxybutyrate metabolism in the present study is not likely to be due to changes in enzyme levels since the expression of β-hydroxybutyrate dehydrogenase determined via immunofluorescence was similar in both TBI and sham brains (Figure 2). To date, many reports, using *in vitro* models and ^13^C or ^14^C labeling techniques, have demonstrated that neurons, oligodendrocytes, and astrocytes are capable of using β-hydroxybutyrate for energy metabolism and synthesis of lipids, and synthesis of neurotransmitters in neurons [20,45,46,47,48,49,50,51,52]. In the present study, histological evaluation showed that β-hydroxybutyrate dehydrogenase was present in both astrocytes and neurons, and no differences were observed in immunofluorescence levels between the TBI and sham rat brain.

Indeed, it is well-established that CNS ketone utilization is highly regulated by the supply from systemic circulation and expression of monocarboxylic acid (MCT) transporters, which serve as carriers for BHB entry into the brain [53,54]. MCT1 mediates uptake of ketones across the blood–brain barrier and uptake by astrocytes, whereas MCT2 mediates uptake by neurons [53]. Expression of these transporters changes during development, with the highest expression of MCT1 and MCT2 at postnatal day 21 in rat brain [26,55]. Following uptake, β-hydroxybutyrate is converted to acetyl-CoA in the mitochondrial matrix via three enzymes: β-hydroxybutyrate dehydrogenase, 3-ketoacid transferase (SCOT), and mitochondrial thiolase (Figure 3A). Interestingly, β-hydroxybutyrate dehydrogenase activity is constant during development; in contrast, the activities of SCOT and mitochondrial thiolase are subject to developmental changes. Specifically, both SCOT and mitochondrial thiolase increase three-to-four-fold after birth, reaching a peak between days 12–20, followed by a decrease in activity in the adult rat brain [56,57]. The enzymes responsible for BHB metabolism are also subject to regional differences in expression [58].

In the present study, metabolism of [2,4-^13^C]β-hydroxybutyrate was increased in brains of TBI rats compared to sham controls. This could be due to increased uptake of BHB into the injured brain after TBI. The finding that there was no change in the level of β-hydroxybutyrate dehydrogenase in the TBI brain is consistent with this concept. Prins and Giza [59] found increased MCT2 expression in the brain after TBI in 35-day-old rats, suggesting that there could be increased neuronal uptake of BHB in the 21-day-old rats in the present study. In the present study, infusion of [2,4-^13^C]β-hydroxybutyrate led to considerably higher incorporation of the label into glutamate than glutamine, and a higher ratio of total labeling in Glu/Gln, indicating that metabolism of BHB was higher in neurons than astrocytes in immature rat brain. These findings are consistent with a report by Roy et al. [60] that the Glu C4/Gln C4 ratio was >2 following the infusion of [2,4-^13^C]β-hydroxybutyrate in 6-week-old rats. Previous *in vitro* studies using primary cultures [45] and cortical brain slices [49] and *in vivo* studies in adult rats [61,62] reported increased incorporation of label from the metabolism of ^13^C-BHB into glutamate compared to glutamine. The *in vivo* studies by Chowdhury et al. [62] provided evidence that ketones, when present in high concentrations, account for ~ 62 percent of substrate oxidation by neurons in the uninjured rat brain. Importantly, a human study by Pan and colleagues demonstrated that the utilization of [2,4-^13^C]β-hydroxybutyrate in the adult brain is primarily neuronal and controlled by uptake at the blood–brain barrier [33]. Our data showing increased metabolism of BHB in young rats after TBI injury indicate that administration of ketones can readily support oxidative metabolism and neurotransmitter synthesis during the timeframe in which brain glucose metabolism is disrupted [11]. Labeling of glutamine from the metabolism of [2,4-^13^C]β-hydroxybutyrate can occur in two ways: by metabolism and direct formation in astrocytes, or by uptake of the ^13^C-glutamate formed in neurons and subsequent conversion to glutamine in astrocytes. Since the ratio of Glu C4/Gln C4 was not significantly changed after TBI, this demonstrates that metabolism of BHB was increased in both neurons and astrocytes in injured brain. Future studies should determine whether prolonged infusions of BHB result in improved bioenergetics in the developing brain. In 17-day-old rats, cerebral glucose metabolism was comparable to sham by 24 h after TBI [10]. Studies using 2-deoxyglucose autoradiography showed that cerebral hypometabolism resolved in young rats earlier than in adult brain after fluid percussion TBI injury [16]. Additional studies aimed to determine whether prolonged exogenous BHB infusion results in changes in expression of genes and proteins responsible for BHB brain uptake and metabolism will provide much needed understanding about dynamic metabolic perturbations after TBI.

In the present study, we observed increased incorporation of the label from the metabolism of [2,4-^13^C]β-hydroxybutyrate into aspartate in TBI brain compared to sham brain. Others reported that incorporation of ^13^C label was increased under hyperketonemic conditions in the rat brain [61,62]. Perez-Liebana showed that treatment with β-hydroxybutyrate led to a three-fold increase in aspartate concentration in neurons from mice with a deficiency of the mitochondrial aspartate-glutamate carrier aralar [63].

The pyruvate recycling pathway leads to formation of pyruvate from TCA cycle intermediates when the generation of pyruvate from glucose metabolism is decreased. This pathway is reported to be present in both astrocytes and neurons; however, the relative cellular activity of this pathway in neurons versus astrocytes remains a subject of debate [35,36,37,64]. This pathway peaks at postnatal day 21 and remains constant thereafter [37,64]. Labeling via this pathway is higher from the metabolism of alternative substrates, including β-hydroxybutyrate, acetate, and acetyl-L-carnitine, than from glucose metabolism [35,36,37,65]. We determined pyruvate recycling by measuring the incorporation of label from the metabolism of [2,4-^13^C]β-hydroxybutyrate into the C3 position of lactate (Lac C3) which can only occur via this pathway. Our results showed that, following TBI in the immature rat brain, the potentially neuroprotective pyruvate recycling was increased in both ipsilateral and contralateral sides of the brain compared to shams.

Our findings that following focal moderate–severe TBI, both the injured (ipsilateral) and uninjured (contralateral) sides of the brain demonstrated increased metabolism of exogenous [2,4-^13^C]β-hydroxybutyrate are consistent with earlier studies, which showed dysregulated glucose metabolism occurs in both the ipsilateral and contralateral sides of the brain [10,11]. Similar findings were reported in adult rat brain following fluid percussion injury [13]. These data suggest that, although there is no overt cell loss in the uninjured (contralateral) side of the brain, it may require metabolic support due to disrupted glucose metabolism after focal TBI.

One limitation of this study is that we included only male rats since, clinically males have a higher incidence of TBI in the young pediatric population. Recently, Greco et al. reported that neither early (first 3 h) nor delayed (6–9 h post-TBI) administration of ketones improved brain bioenergetics in young adult female rats [32]. Future experiments should include animals of both sexes to delineate whether younger brains exhibit sex differences in metabolism after injury.

Overall, the most important findings from our study are: (1) the immature brain has the capacity to increase oxidative metabolism when provided with ketones which bypass metabolism at the PDH pathway; (2) this increased metabolism occurs in both astrocyte and neuron TCA cycles; (3) the capacity for pyruvate recycling increases after TBI in the immature brain. The results of this preclinical study provide direct evidence that ketones can readily be utilized early after TBI in the immature brain and thus may represent a promising therapeutic intervention clinically. Further studies are needed to delineate the duration of increased β-hydroxybutyrate metabolism in young animals after TBI in a time-, region-, and cell-specific manner and determine whether exogenous administration of BHB may serve as a therapeutic intervention to support brain oxidative metabolism.

## Figures and Tables

**Table 1:** Blood levels of glucose and β-hydroxybutyrate (BHB).

Time	Group	Glucose (mmol/L)	BHB (mmol/L)
0 h	Sham + NS	14.87 ± 1.12	0.43 ± 0.11
	Sham + BHB	11.28 ± 0.86	0.45 ± 0.12
	TBI + NS	16.53 ± 3.46	0.70 ± 0.19
	TBI + BHB	14.85 ± 1.73	0.80 ± 0.21
1 h	Sham + NS	22.03 ± 3.54	0.37 ± 0.04
	Sham + BHB	13.70 ± 2.67	0.57 ± 0.12
	TBI + NS	22.33 ± 2.76	0.80 ± 0.25
	TBI + BHB	14.37 ± 6.45	0.87 ± 0.26
1 h 20 min	Sham + NS	13.55 ± 4.05	0.60 ± 0.28
	Sham + BHB	10.73 ± 1.25	1.76 ± 0.25
	TBI + NS	16.80 ± 1.80	0.83 ± 0.21
	TBI + BHB	14.93 ± 6.53	1.87 ± 0.54
4 h	Sham + NS	9.83 ± 2.21	0.57 ± 0.11
	Sham + BHB	10.08 ± 3.08	2.15 ± 0.76
	TBI + NS	10.03 ± 1.21	0.40 ± 0.10
	TBI + BHB	8.10 ± 1.92	2.65 ± 1.20

**Table 2:** Ratios of metabolite labeling and cycling.

Ratios	Sham Ipsi	Sham Contra	TBI Ipsi	TBI Contra
**Metabolite ratios**				
Glu C4/Gln C4	2.12 ± 0.09	2.08 ± 0.1	2.36 ± 0.09	2.50 ± 0.15
Asp C3/Glu C4	0.14 ± 0.01	0.17 ±0.02	0.13 ± 0.01	0.14 ± 0.01
Total GABA/Total Glu	0.20 ± 0.02	0.20 ± 0.03	0.15 ± 0.01	0.16 ± 0.02
**Cycling ratios**				
Glu C3/Glu C4	0.77 ± 0.07	0.87 ± 0.08	0.66 ± 0.03	0.67 ± 0.15
GABA C3/GABAC2	0.91 ± 0.06	1.06 ± 0.06	0.76 ± 0.08	0.90 ± 0.09
Gln C3/Gln C4	0.57 ± 0.04	0.62 ± 0.03	0.48 ± 0.03	0.54 ± 0.05
TCA cycling $\frac{Glu\;\left(C2+C3\right)}{Glu\;C4}$	1.43 ± 0.13	1.55 ± 0.14	1.30 ± 0.06	1.28 ± 0.11

## Data Availability

Data are contained within the article.

## Supplementary Materials

The following supporting information can be downloaded at: https://www.mdpi.com/article/10.3390/metabo12080710/s1, Figure S1: Plasma spectra.
