# Supplementary material for: Metabolism of Exogenous [2,4-13C]β-Hydroxybutyrate following Traumatic Brain Injury in 21-22-Day-Old Rats: An Ex Vivo NMR Study
Source: Metabolites. 2022 Jul 29;12(8):710. doi: 10.3390/metabo12080710 (PMC9414923; doi:10.3390/metabo12080710)
Supplement: Supplementary file 1 [file metabolites-12-00710-s001.zip › metabolites-1796660-supplementary.pdf]

metabolites

MDPI

Scafidi et al. Supplemental Figure S1.

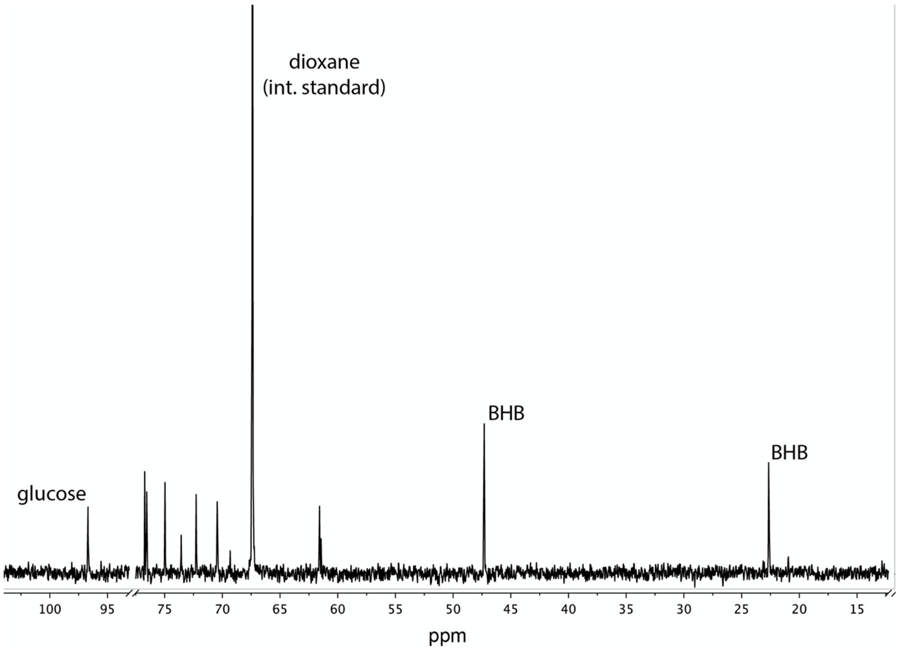

Supplemental Figure S1. Representative plasma spectra following the infusion of [2,4-^{13}C] β-hydroxybutyrate. Abbreviations: BHB = β-hydroxybutyrate.
